# Efficacy and Pulmonary Outcomes of Dual Immunotherapy Plus Chemotherapy Versus Standard Chemotherapy in Advanced Non-small Cell Lung Cancer

**DOI:** 10.7759/cureus.98184

**Published:** 2025-11-30

**Authors:** Bryan Nicolás Forero Vásquez, Alexis Agustin A Dunay Silva, Juan Sebastian Hernández Cedeño, Diego Alejandro Salinas Lucero, Gustavo Andres Acevedo Carrion, Juan Felipe Perez-Correa, Isaac Baruch Salazar Nieto

**Affiliations:** 1 Internal Medicine, Fundación Hospital San Carlos, Bogotá, COL; 2 Internal Medicine, Hospital Barros Luco Trudeau, Santiago, CHL; 3 General Medicine, Universidad Católica Santiago de Guayaquil, Guayaquil, ECU; 4 Epidemiology, Universidad de Tolima, Ibague, COL; 5 Internal Medicine, Colombian Medical College, Ubate Cundinamarca, COL; 6 Medicine, Universidad del Rosario, Bogotá, COL; 7 Internal Medicine, Mexican Social Security Institute, Monterrey, MEX

**Keywords:** cancer immunotherapy, chemotherapy, efficacy, non-small cell lung cancer, systematic review

## Abstract

Advanced non-small cell lung cancer (NSCLC) presents significant therapeutic challenges, with limited long-term survival using conventional chemotherapy. This review aims to evaluate the efficacy and pulmonary safety of dual immunotherapy (nivolumab, ipilimumab, tremelimumab, and durvalumab) plus chemotherapy compared with standard chemotherapy in advanced NSCLC. This Preferred Reporting Items for Systematic Reviews and Meta-Analyses (PRISMA)-based systematic review searched PubMed, Cochrane Library, and ScienceDirect (from inception till June 2025) using MeSH/free-text terms for “NSCLC,” “dual immunotherapy,” “checkpoint inhibitor,” “chemotherapy,” and “pulmonary outcomes.” Two reviewers screened studies, extracted data, and assessed risk of bias with Cochrane RoB 2.0. Phase II/III randomized controlled trials (RCTs) comparing dual immunotherapy plus chemotherapy versus chemotherapy were narratively synthesized for survival and lung-related outcomes due to endpoint heterogeneity. Nine studies, including 7,271 participants with advanced or metastatic NSCLC, were analyzed. Dual immunotherapy plus chemotherapy consistently improved outcomes compared with chemotherapy alone. Median overall survival (OS) increased to approximately 15.8 months versus 11.0 months, with hazard ratios (HRs) ranging from 0.66 to 0.77. Progression-free survival (PFS) and response rates (HRs) were also higher, with pooled PFS HRs of 0.67-0.72 and longer duration of response. Pulmonary safety was acceptable, with immune-related pneumonitis reported in 2%-5% of patients, mostly low-grade and responsive to corticosteroids. Infection rates were comparable between groups, primarily involving mild respiratory infections typical of chemotherapy exposure. Survival benefits were observed across PD-L1 strata, tumor histologies, and high-risk molecular subgroups such as KRAS, STK11, and KEAP1, and in patients with brain metastases. Dual immunotherapy plus chemotherapy provides sustained survival benefit with manageable toxicity. Dual immunotherapy plus chemotherapy significantly improves survival and response in advanced NSCLC with acceptable pulmonary safety, supporting its adoption as a first-line standard of care.

## Introduction and background

Lung cancer is the malignant tumor with the highest morbidity and mortality in the world, and approximately 85% of patients with lung cancer have non-small cell lung cancer (NSCLC) [1, 2]. NSCLC includes squamous NSCLC and non-squamous NSCLC, among which non-squamous NSCLC is more common [3]. Almost 70% of NSCLC cases have spread to local or distant sites at the time of diagnosis and are diagnosed with locally advanced or advanced stage due to atypical symptoms in the early stage [4,5]. Historically, platinum-based chemotherapy was the standard first-line therapy for advanced NSCLC, providing modest improvement in survival and limited duration of disease control. However, it is also associated with substantial acute and chronic toxicity, including adverse effects on lung function, especially in patients with underlying pulmonary compromise.

In recent years, immune checkpoint inhibitors (ICIs) targeting PD-1, PD-L1, and CTLA-4 have transformed the therapeutic landscape. Monotherapy with PD-1/PD-L1 inhibitors improves outcomes in patients with high PD-L1 expression, and combinations of ICI + chemotherapy have become standard in many settings [6]. More recently, dual immunotherapy, blocking more than one checkpoint, such as PD-1 plus CTLA-4, has gained attention following results from landmark trials including CheckMate 9LA, POSEIDON, and CheckMate 227. These regimens seek to harness the synergistic effects of dual checkpoint inhibition while limiting chemo-related toxicity by reducing the number of chemotherapy cycles [7]. ICIs act by blocking inhibitory pathways that tumors exploit to evade immune destruction. In NSCLC, PD‐1 on activated T cells binds PD‐L1 (on tumor or stromal cells), suppressing T‐cell proliferation, cytokine release, and cytotoxic function; antibodies targeting PD‐1 or PD‐L1 release this “brake,” restoring anti‐tumor T cell activity [8]. CTLA-4 inhibition (e.g., ipilimumab, tremelimumab) enhances early T-cell priming, but a concise immunologic summary highlights that dual blockade broadens T-cell activation across multiple stages of the anti-tumor response. This combined pathway activation increases tumor-infiltrating lymphocytes, enhances clonal diversity, and may overcome microenvironment-driven resistance mechanisms [9,10].

Recent systematic reviews have compared different immunotherapy-based regimens in advanced NSCLC. For example, a network meta-analysis in 2021 found that among immunotherapy combinations, regimens such as pembrolizumab + chemotherapy and atezolizumab + bevacizumab + chemotherapy showed strong OS and PFS advantages, especially in PD-L1-selected populations [11]. Another narrative review in 2025 emphasizes that while immunotherapy combinations are promising, real-world lung toxicities and long-term pulmonary function outcomes remain underreported [12]. Many previous systematic reviews compared immunotherapy (mainly single-agent checkpoint inhibitors) with chemotherapy, showing survival gains but limited safety data [6,13,14]. However, previous systematic reviews have primarily compared single-agent immunotherapy with standard chemotherapy, and to date, no review has specifically evaluated dual immunotherapy plus chemotherapy versus standard chemotherapy, leaving a critical gap in understanding the added benefit and unique toxicity profile of these regimens. Understanding this evidence is essential for clinical decision-making, as clinicians must balance survival gains with the risk of immune-related pneumonitis and infection, and policymakers increasingly rely on comparative efficacy-toxicity profiles when shaping reimbursement and treatment guidelines. This review, therefore, synthesizes evidence on survival, pulmonary outcomes, and safety of dual immunotherapy plus chemotherapy versus standard chemotherapy, addressing an urgent need for integrated clinical and policy-relevant data on these emerging regimens.

## Review

Methods

Study Design

This study is a systematic review conducted in accordance with the Preferred Reporting Items for Systematic Reviews and Meta-Analyses (PRISMA) guidelines [15]. The objective was to evaluate and compare the efficacy and pulmonary safety outcomes of dual immunotherapy plus chemotherapy versus conventional chemotherapy in patients with advanced NSCLC. Primary outcomes of interest were overall survival (OS) and pulmonary/lung-related outcomes, including immune-related pneumonitis and long-term safety signals.

Search Strategy

A comprehensive literature search was performed across PubMed, Cochrane Library, and ScienceDirect, covering publications from database inception to June 2025. The following MeSH terms and free-text keywords were used in various combinations: “non-small cell lung cancer,” “NSCLC,” “advanced NSCLC,” “immunotherapy,” “dual immunotherapy,” “checkpoint inhibitor,” “nivolumab,” “ipilimumab,” “durvalumab,” “tremelimumab,” “atezolizumab,” “chemotherapy,” “overall survival,” “lung function,” “pneumonitis,” and “immune-related adverse events”. Boolean operators “AND” and “OR” were applied to refine the search. Detailed search strategy is listed in Appendix A. The reference lists of relevant articles and prior meta-analyses were also manually screened to identify additional eligible studies.

Study Selection

All identified records were screened by title and abstract. Full-text articles were then retrieved for potentially relevant studies. A PRISMA flow diagram was constructed to illustrate the study selection process [15].

Eligibility Criteria

The eligibility criteria for this review were defined using the Population, Intervention, Comparison, Outcomes, and Study (PICOS) framework. Studies were included if they involved adults (≥18 years) diagnosed with advanced or recurrent NSCLC and evaluated dual ICI therapy, such as nivolumab plus ipilimumab or durvalumab plus tremelimumab, in combination with chemotherapy. The comparator was conventional chemotherapy with or without maintenance therapy. Eligible studies were required to report at least one key outcome, including OS, progression-free survival (PFS), objective response rate (ORR), duration of response (DOR), or pulmonary outcomes such as lung function or immune-mediated pulmonary adverse events. Only Phase II or III randomized controlled trials (RCTs) or comparative clinical studies with extractable data were included. Excluded were review papers, meta-analyses, case reports, abstracts lacking data, retrospective studies, preclinical research, editorials, letters, and studies without relevant outcomes.

Data Extraction

Data extraction was performed independently by two reviewers using a standardized template. Extracted information included first author and year, trial name/design, study population and sample size, intervention and control arms, primary and secondary endpoints, OS and PFS data, ORR, DOR, and safety outcomes with emphasis on pulmonary toxicity (e.g., pneumonitis, interstitial lung disease). Where reported, subgroup analyses (PD-L1 status, histology, mutation status, and brain metastases) were also recorded. Any discrepancies between reviewers were resolved through discussion and consensus, and when needed, a third reviewer adjudicated remaining disagreements.

Quality Assessment

The methodological quality and risk of bias of included RCTs were assessed using the Cochrane Risk of Bias 2.0 (RoB 2.0) tool [16], a freely available standardized tool that evaluates domains such as randomization, deviations from intended interventions, missing outcome data, measurement of outcomes, and selective reporting.

Data Synthesis

A narrative synthesis was conducted across all included phase II/III randomized trials evaluating dual immunotherapy plus chemotherapy versus standard chemotherapy in advanced NSCLC. Study findings were qualitatively summarized across five predefined domains: OS outcomes; PFS and response rates (ORR, DOR); pulmonary and general safety outcomes; consistency across clinically relevant subgroups; and long-term implications. A formal meta-analysis was not performed due to substantial clinical and methodological heterogeneity among the included trials, including differences in immunotherapy regimens (nivolumab + ipilimumab vs. tremelimumab + durvalumab), number of chemotherapy cycles, chemotherapy backbones, patient characteristics (e.g., PD-L1 expression, EGFR/ALK status, histology, brain metastases), and variable outcome reporting formats and follow-up durations. Given these sources of clinical and statistical heterogeneity, a qualitative synthesis was the most appropriate approach to accurately summarize comparative efficacy, safety, and subgroup patterns across trials.

Results

**Figure 1:**
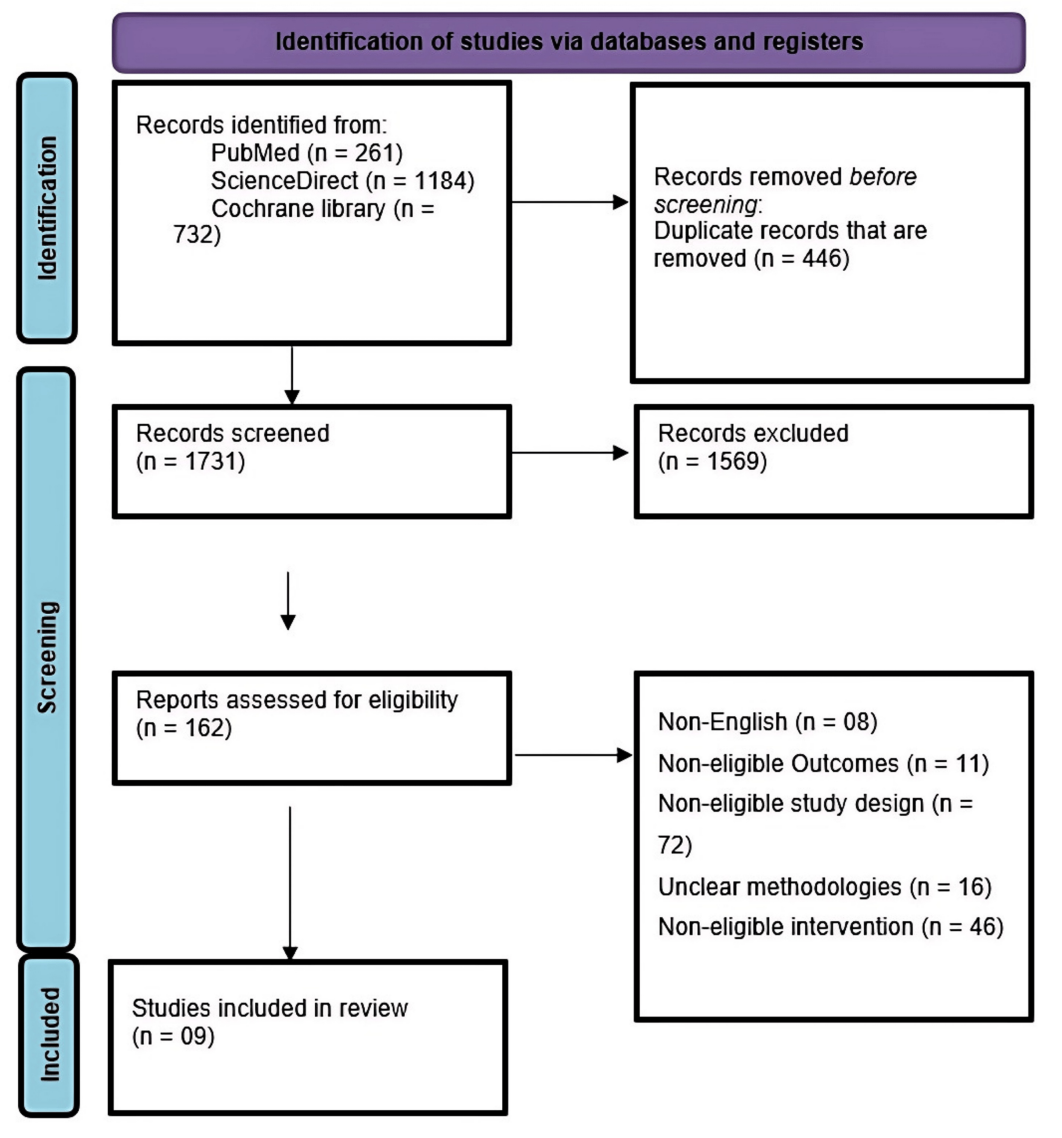
A PRISMA flow diagram outlining the study selection process PRISMA: Preferred Reporting Items for Systematic Reviews and Meta-Analyses  [15]

A total of 2,177 records were retrieved from three databases: PubMed (n = 261), ScienceDirect (n = 1,184), and the Cochrane Library (n = 732). After removing 446 duplicate records, 1,731 studies remained for screening based on titles and abstracts. Of these, 1,569 were excluded for not meeting the eligibility criteria. The remaining 162 full-text articles were assessed for eligibility, with exclusions due to non-English language (n = 8), “non-eligible outcomes (n = 11), unsuitable study design (n = 72), unclear methodologies (n = 16), and incompatible interventions (n = 46). Ultimately, nine studies fulfilled the inclusion criteria and were included in the final qualitative synthesis (Figure 1). 

Study Characteristics

The systematic review included nine Phase III RCTs published between 2021 and 2025, evaluating first-line immunotherapy-based regimens for advanced or metastatic NSCLC. Across studies, sample sizes ranged from 637 to 1,013 participants, predominantly involving patients with metastatic or recurrent NSCLC without EGFR or ALK alterations and with Eastern Cooperative Oncology Group (ECOG) performance status 0-1. Several trials assessed dual immune checkpoint blockade with nivolumab plus ipilimumab combined with two cycles of platinum-doublet chemotherapy (CheckMate 227/9LA series), while others investigated tremelimumab plus durvalumab with chemotherapy (T+D+CT) or durvalumab plus chemotherapy (D+CT) alone (POSEIDON program). Comparator arms consistently involved standard platinum-based chemotherapy for up to four cycles, with pemetrexed use per histologic subtype. The primary endpoints across studies were OS and PFS, with additional outcomes including ORR, DOR, and safety. Follow-up durations ranged from 24 to 76 months, enabling long-term survival assessment, including several analyses reporting outcomes beyond four to six years. Although no study directly evaluated pulmonary or lung-function parameters, select trials reported immune-related pulmonary toxicities such as pneumonitis, typically occurring at low frequencies. PD-L1 subgroup analyses were widely incorporated, with consistent efficacy observed in both PD-L1 <1% and ≥1% cohorts. Several studies also reported survival benefits across key molecular subgroups, including KRAS, STK11, KEAP1, and TP53 mutations, and in patients with brain metastases. Key characteristics and findings are summarized in Table 1. 

**Table 1:** Characteristics and main findings of the studies included NSCLC: non–small cell lung cancer; Chemo: chemotherapy; OS: overall survival; PFS: progression-free survival; ORR: objective response rate; DOR: duration of response; Mo: months; Y: years; T+D+CT: tremelimumab plus durvalumab plus chemotherapy; D+CT: durvalumab plus chemotherapy; HR: hazard ratio; RCT: randomized controlled trial; TRAE: treatment-related adverse event; ECOG: Eastern Cooperative Oncology Group; AEs: adverse events

Study Author/Year	Study Design	Population (N)	Intervention Arm(s)	Control Arm(s)	Primary Endpoint(s)	OS Outcomes	Pulmonary/Lung Function Outcomes	Follow-up Duration	PD-L1 Subgroup Analysis	Other Findings
Peters et al., 2024 [17]	Randomized phase 3 pooled (CheckMate 227 & 9LA)	637 metastatic NSCLC, PD-L1 <1%	Nivolumab + ipilimumab ± 2 cycles chemo	Chemo up to four cycles	OS, PFS, ORR, DOR, safety	Median OS 17.4 vs 11.3 mo; 5-y OS 20% vs 7%	Lung benefit inferred via durable survival in squamous and nonsquamous subtypes	5 years	Benefit consistent in PD-L1 <1%	Improved PFS (5.4 vs 4.9 mo), ORR (29% vs 22%), DOR 18 vs 4.6 mo; benefit maintained in brain metastases; safety manageable.
Peters et al., 2025 [18]	Randomized phase 3, open-label, global trial (POSEIDON)	1013 EGFR/ALK WT metastatic NSCLC	T+D+CT; D+CT	CT	OS, PFS	T+D+CT improved OS (HR 0.76, 95% CI 0.65–0.88), and D+CT showed a smaller benefit (HR 0.84, 95% CI 0.71–0.99).	No direct pulmonary outcomes assessed	63.4 months	Efficacy consistent across PD-L1 subgroups	Durable OS benefit in nonsquamous, STK11, KRAS, and KEAP1 mutation subsets; safety acceptable.
Garon et al., 2024 [19]	Phase 3 RCT (POSEIDON Brief)	1013 mNSCLC, EGFR/ALK WT	T+D+CT; D+CT	Chemo	OS, PFS	T+D+CT improved OS (HR 0.77, 95% CI 0.65–0.90), with median OS 12.7–15.6 vs 11.0–12.5 months.	Grade ≥3 pneumonitis/ILD <2%	46.5 months	Benefit maintained across PD-L1 <1% and ≥1%	Improved PFS and ORR; benefit across histology; D+CT improved PFS, OS trend non-significant; safety acceptable.
Carbone et al., 2024 [20]	Phase III RCT (CheckMate 9LA)	719 stage IV/recurrent NSCLC, ECOG ≤1	Nivolumab + ipilimumab + 2 cycles chemo	Chemo ± pemetrexed	OS	Median OS 15.8 vs 11.0 mo; 4-yr OS 21% vs 16%	Not directly measured	4 years	Consistent across PD-L1 subgroups	Durable survival across histologies; OS HR adjusted to 0.66 for crossover; PFS HR 0.70; TRAE discontinuation 4-yr OS 41%.
Paz-Ares et al., 2021 [21]	Phase III RCT (CheckMate 9LA)	719 advanced NSCLC, EGFR/ALK–, ECOG ≤1	Nivolumab + Ipilimumab + 2 cycles chemo	4 cycles chemo ± pemetrexed	OS	Median OS 15.8 vs 11.0 months (HR 0.72, 95% CI 0.61–0.85), with two-year OS of 38% vs 26%.	Pneumonitis noted among TRAEs	30.7 months	Benefit consistent across PD-L1/histology	PFS HR 0.67, ORR 38% vs 25%, DOR 13 vs 5.6 mo; CNS metastases included; TRAEs manageable; discontinuation did not compromise efficacy.
Johnson et al., 2023 [22]	Phase III RCT, open-label	1,013 EGFR/ALK WT mNSCLC	T+D+CT; D+CT	Chemo	PFS, OS	T+D+CT median OS 14.0 months (HR 0.77, 95% CI 0.65–0.91); D+CT 13.3 months (HR 0.86, 95% CI 0.73–1.01).	Pulmonary AEs: grade 3/4 pneumonitis 10% vs 1.5%	24 months	Not reported	T+D+CT improved PFS (6.2 vs 4.8 mo) and OS; D+CT improved PFS with OS trend; safety was manageable.
Carbone et al., 2025 [23]	Phase III RCT, open-label (CheckMate 9LA)	719 NSCLC	Nivolumab + ipilimumab + 2 cycles chemo	Chemo ± pemetrexed	OS	HR 0.74 (95% CI 0.62–0.88); six-yr OS 16% vs 10%.	Not directly measured	6 years	Benefit across PD-L1 <1% and ≥1%	PFS 9% vs 3% (HR 0.70); DOR 19% vs 0%; benefit consistent across KRAS, STK11, KEAP1, TP53 mutations; no new safety signals.
Reck et al., 2021 [24]	Phase III RCT, open-label	719 advanced NSCLC, EGFR/ALK–, ECOG ≤1	Nivolumab + ipilimumab + 2 cycles chemo	4 cycles chemo ± pemetrexed	OS	Median OS 15.8 vs 11.0 mo; two-yr OS 38% vs 26%	Lung function preserved; immune-related AEs manageable	30 months	Consistent across PD-L1/histology	PFS HR 0.67, ORR 38% vs 25%, PFS2 13.9 vs 8.7 mo; 34% vs 12% ongoing responses at 2 yrs; early grade 3/4 AEs; discontinuation median OS 27.5 mo.
Vellanki et al., 2021 [25]	Phase 3 RCT, open-label (CheckMate 9LA)	719 metastatic/recurrent NSCLC	Nivolumab + ipilimumab + 2 cycles chemo	Four cycles of chemo	OS, PFS, ORR	Median OS 14.1 vs 10.7 months (HR 0.69, 95% CI 0.55–0.86); updated 15.6 vs 10.9 months (HR 0.66, 95% CI 0.52–0.82).	Not directly measured; benefit inferred from NSCLC outcomes	Median 36 months	Not reported	PFS 6.8 vs 5.0 mo; ORR 37.7% vs 25.1%; DOR 10 vs 5.1 mo; regimen reduced early mortality and chemo burden; safety manageable.

Quality Assessment

**Figure 2:**
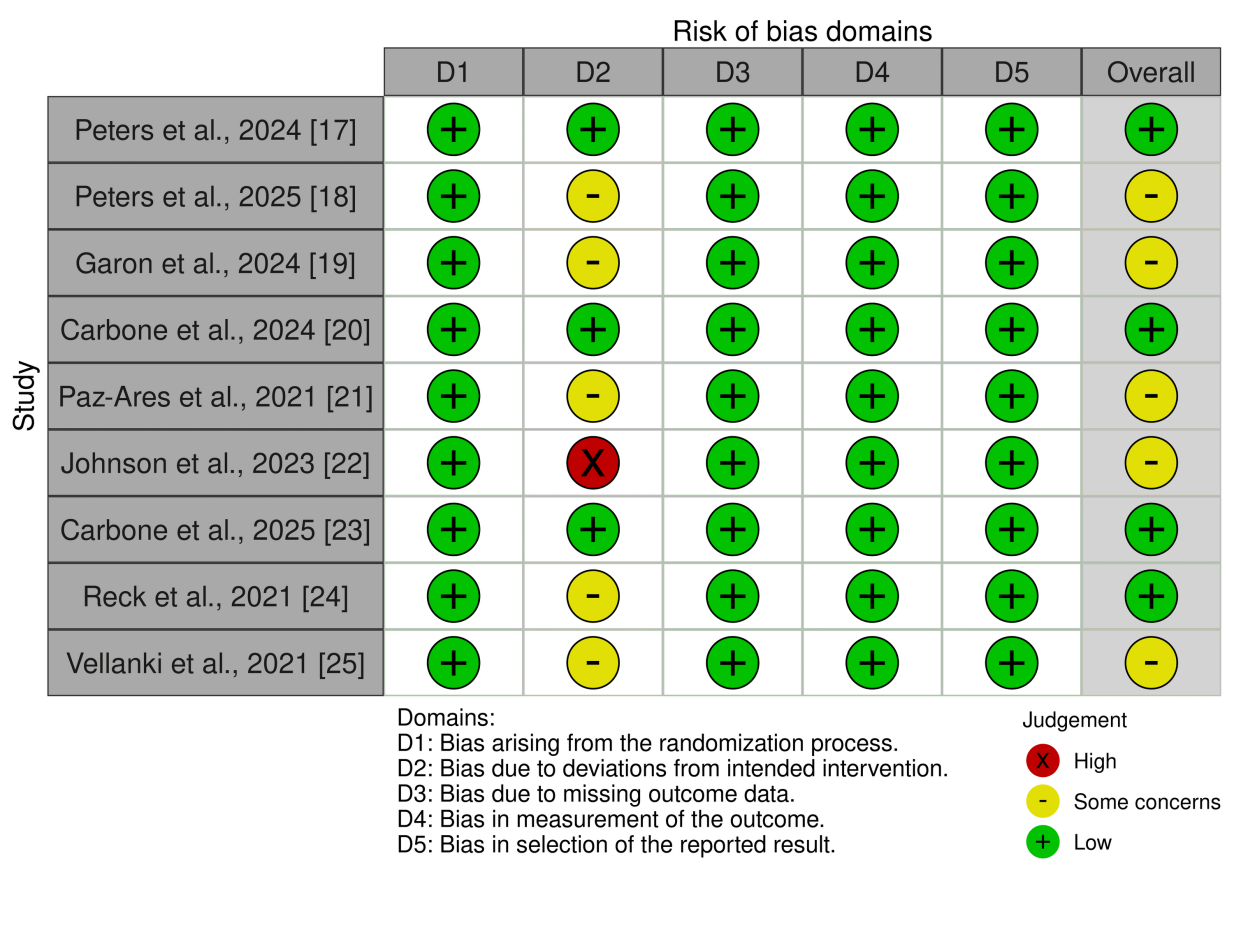
Quality assessment of RCTs by the RoB 2.0 tool RoB 2.0: Cochrane Risk of Bias 2.0 [16].

Across included RoB 2.0 assessments showed predominantly low risk in randomization, missing data, and measurement, with only “some concerns” for deviations and selective reporting, indicating overall high methodological quality and reliability of results (Figure 2).

OS Outcomes

Across all included phase III trials, dual immunotherapy plus chemotherapy demonstrated a consistent survival advantage over standard chemotherapy in patients with advanced NSCLC. In the CheckMate 9LA study, nivolumab plus ipilimumab with two cycles of chemotherapy improved median OS to 15.8 months versus 11.0 months with chemotherapy alone (hazard ratio (HR) 0.74, 95% CI 0.63-0.87), with durable benefits at four and six years [20, 23, 24]. The six-year OS remained higher in the experimental arm (16% vs 10%), including subgroups with PD-L1 <1% (20% vs 7%) and ≥1% (15% vs 10%). Importantly, OS benefit persisted even in patients who discontinued treatment due to adverse events, with survival rates of 34% at six years. The POSEIDON trial further reinforced these findings. T+D+CT significantly prolonged OS compared to chemotherapy alone (HR 0.76, 95% CI 0.64-0.89), with a five-year OS of 15.7% versus 6.8% [17]. D+CT also showed benefit, though less pronounced (HR 0.84). Subgroup analyses demonstrated robust efficacy across PD-L1 subgroups, nonsquamous histology, and high-risk molecular subsets such as STK11 and KRAS mutations. Long-term pooled analyses, including CheckMate 227 and 9LA, confirmed the durability of dual immunotherapy plus chemotherapy, with a five-year OS of 20% compared with 7% for chemotherapy alone [18]. Collectively, these findings establish a sustained OS advantage across histologies and biomarker-defined populations.

PFS and Response Rates

PFS and response rates were also consistently superior in immunotherapy-containing regimens. In CheckMate 9LA, median PFS was 6.8 months with nivolumab plus ipilimumab plus chemotherapy versus five months with chemotherapy (HR 0.70, p=0.0001), with durable responses sustained over four years [24, 25]. At six years, the PFS rate remained 9% with immunotherapy versus 3% with chemotherapy [23]. The POSEIDON trial further demonstrated median PFS improvements (6.2 vs 4.8 months; HR 0.72) with the tremelimumab-containing triplet [22]. ORRs were also higher in immunotherapy groups, such as 37.7% vs 25.1% in CheckMate 9LA and up to 40% vs 28% in POSEIDON, depending on PD-L1 subgroup [19]. The DOR was prolonged as well, with a median DOR exceeding 10 months compared to approximately five months with chemotherapy alone [25].

Pulmonary and Safety Outcomes

Direct pulmonary function measures (e.g., spirometry or lung volumes) were not reported in the included RCTs; therefore, pulmonary safety was assessed through respiratory immune-mediated adverse events. Across CheckMate 9LA, immune-mediated pneumonitis occurred in approximately 4%-5% of patients, and events were generally low-grade and responsive to corticosteroids [23, 24]. Long-term follow-up did not demonstrate an excess of late-onset pulmonary toxicity compared with chemotherapy, indicating no sustained decline in lung function. In POSEIDON, grade ≥3 pulmonary adverse events were uncommon, with pneumonitis rates of 2%-3%, slightly higher than chemotherapy alone but remaining clinically manageable [19, 22]. Safety analyses across trials confirmed that discontinuation of therapy due to treatment-related adverse events (TRAEs) did not compromise long-term efficacy; for example, patients who discontinued immunotherapy in CheckMate 9LA still achieved a 34% overall survival at six years [23]. Overall, dual immunotherapy plus chemotherapy demonstrated a manageable pulmonary safety profile without evidence of additive long-term lung toxicity.

Consistency Across Subgroups and Long-Term Implications

The OS and PFS benefits of dual immunotherapy plus chemotherapy were consistent across patient subgroups, including those with squamous and non-squamous histology, PD-L1 negative tumors, and patients with brain metastases. For example, CheckMate 9LA reported HRs of 0.51 for squamous histology and 0.44 for brain metastases [17]. Genetic subsets, including patients with KRAS, STK11, and KEAP1 mutations, also derived meaningful survival benefits [18]. This consistency can be biologically explained by the complementary mechanisms of dual immune checkpoint blockade: PD-1/PD-L1 inhibition restores cytotoxic T-cell activity, while CTLA-4 inhibition enhances T-cell priming and broadens the tumor-infiltrating lymphocyte repertoire. By engaging multiple steps of the anti-tumor immune response, dual blockade may overcome intrinsic tumor heterogeneity, low PD-L1 expression, and immunosuppressive microenvironments, thus producing efficacy across histologic, molecular, and CNS-involved subgroups. The durability of long-term survival, with up to 20% of patients alive at five years and 16% at six years, underscores the potential of this regimen as a new first-line standard, with survival advantages outweighing manageable pulmonary and immune-related risks.

Discussion

The results of this systematic review demonstrate that dual immune checkpoint inhibition combined with chemotherapy yields a consistent and clinically meaningful survival benefit over conventional chemotherapy in patients with advanced NSCLC. Across multiple phase III randomized trials, such as CheckMate 9LA, POSEIDON, and the pooled analysis of CheckMate 227/9LA, combined regimens (e.g., nivolumab + ipilimumab + a short course of chemotherapy; durvalumab + tremelimumab + chemotherapy) improved median OS, PFS, ORR, and DOR compared to chemotherapy alone. For example, in CheckMate 9LA, the median OS rose from ~11.0 months with chemotherapy alone to ~15.8 months with the dual immunotherapy plus chemotherapy regimen; similarly, five- or six-year survival rates more than doubled in many studies [17]. These benefits appear robust across subgroups: patients with low PD-L1 (<1%) expression, differing histologies (squamous and nonsquamous), and even high-risk molecular subsets such as KRAS, STK11, and KEAP1 mutation carriers all exhibited improved survival with dual immunotherapy plus chemo vs. standard chemotherapy [23, 22]. Our findings align closely with the updated meta-analysis by Chai et al. (2022) [13], which reported significantly improved ORR (OR 2.81), PFS (HR 0.63), and OS (HR 0.68) for immunotherapy plus chemotherapy versus chemotherapy alone in PD-L1-negative, driver-gene-negative nonsquamous NSCLC [13]. Similar to their results, we observed consistent survival benefits, albeit with higher immune-related adverse events.

In addition to survival metrics, response and disease control were superior in combined therapy arms. PFS was consistently longer, ORR higher, and DOR extended in the dual therapy arms relative to chemotherapy alone. Notably, POSEIDON and CheckMate 9LA reported response durability that persisted in long-term follow-up, with maintained disease control beyond one or two years in a subset of patients [17,18]. These gains in disease control translate into meaningful clinical benefit: a greater proportion of patients experience prolonged life, and many avoid early progression, which often entails symptomatic decline. Together, these efficacy findings suggest that dual immunotherapy added to chemotherapy can shift the prognosis for advanced NSCLC, particularly among populations who otherwise derive limited benefit from chemotherapy alone. Our results mirror those of Abdelazeem et al. (2022), whose meta-analysis of 11 RCTs (n=6,386) showed significant gains in PFS (HR 0.60) and OS (HR 0.77) for immunotherapy plus chemotherapy versus chemotherapy alone, without a significant rise in overall TRAEs (RR 1.07). Likewise, our review found durable response and survival benefits with manageable toxicity [7].

Pulmonary safety and tolerability are critical concerns when combining immune checkpoint inhibitors, particularly dual inhibition, with chemotherapy. In the reviewed trials, immune-related pneumonitis and other lung toxicities (e.g., interstitial lung disease) occurred more frequently in the combined therapy arms compared to chemotherapy, but rates of severe (grade ≥3) pulmonary adverse events were generally low (often ~2%-5%), manageable, and rarely fatal [19, 23, 22]. Direct lung function testing (spirometry, diffusing capacity for carbon monoxide) was seldom included, limiting assessment of subclinical or long-term pulmonary impairment, but long-term follow-up data do not suggest excessive late-onset pulmonary decline beyond that expected with immunotherapy or chemotherapy alone. Thus, while pulmonary risk is nontrivial, it appears acceptable in light of survival gains for most trial populations. Unlike Abdelazeem et al.’s pooled data, our review observed a modestly higher incidence of grade ≥3 pneumonitis (approximately 2%-5%) [7]. However, these events remained clinically manageable with established protocols and did not outweigh the survival and disease control benefits of dual immunotherapy plus chemotherapy, supporting its favorable overall risk-benefit profile in advanced NSCLC.

There are several limitations to the available evidence and to this review. First, the heterogeneity of included trials, in terms of chemotherapy regimens, number of chemotherapy cycles, specific immunotherapy agents, duration of follow-up, and eligibility criteria (e.g., excluding patients with severe underlying lung disease), makes direct comparison and generalization challenging. Second, the relative absence of objective lung function endpoints and respiratory quality-of-life measures reduces our ability to fully understand the functional pulmonary impact of dual immunotherapy plus chemotherapy, particularly in patients with baseline pulmonary compromise. Third, many trials excluded or underrepresented patients with comorbidities or real-world frailty, creating potential bias toward more favorable outcomes. Finally, publication bias or selective reporting of adverse events could underestimate lung‐related harms. From a clinical standpoint, these findings support considering dual immunotherapy plus chemotherapy as a first-line option for fit patients with advanced NSCLC, especially those without driver mutations, even in PD-L1 low expressers. Shared decision-making should include discussion of pulmonary risks, monitoring strategies, and patient priorities (e.g., quality of life, functional capacity). For policymakers, evidence suggests that investing in access to dual immunotherapy regimens may improve population survival. However, cost, resource constraints, and infrastructure for managing immune adverse events, including chest imaging and pulmonary specialist follow-up, are important considerations. Future trials should include standardized lung function monitoring, more representative patient populations, and longer follow-up focused on both survival and respiratory morbidity to better capture long-term safety and quality of life.

## Conclusions

Dual immunotherapy combined with chemotherapy provides superior overall and progression-free survival compared with standard chemotherapy in advanced NSCLC, with durable responses across patient subgroups. Pulmonary adverse events, such as pneumonitis, represent expected immune-related effects of checkpoint inhibition rather than unexpected toxicities; they occur more frequently than with chemotherapy alone but are mostly low-grade, manageable, and rarely fatal. Limited direct lung-function data highlight the need for longer follow-up to characterize pulmonary safety fully. Overall, the evidence supports dual immunotherapy plus chemotherapy as a promising first-line approach, with ongoing research warranted to clarify long-term pulmonary outcomes and quality-of-life impacts.

## Appendices

Appendix A

Detailed Search Strategies

PubMed: ("Non-Small Cell Lung Cancer"[Mesh] OR "NSCLC" OR "non small cell lung cancer" OR "lung carcinoma, non-small-cell") AND ( ("nivolumab" OR "Opdivo") AND ("ipilimumab" OR "Yervoy") OR ("durvalumab" OR "Imfinzi") OR ("tremelimumab") ) AND ("chemotherapy" OR "platinum-based chemotherapy" OR "pemetrexed" OR "cisplatin" OR "carboplatin" OR "platinum doublet") AND ("randomized controlled trial"[pt] OR "randomized"[tiab] OR "phase III"[tiab] OR "phase 3"[tiab] OR "clinical trial"[pt])

ScienceDirect: ("non small cell lung cancer" OR NSCLC) AND (nivolumab AND ipilimumab OR durvalumab OR tremelimumab) AND (chemotherapy OR "platinum chemotherapy OR cisplatin OR carboplatin)

Cochrane Library: ("non small cell lung cancer" OR NSCLC) AND (nivolumab OR ipilimumab OR durvalumab OR tremelimumab) AND (chemotherapy) ("phase III" OR "phase 3" OR randomized OR randomised)
